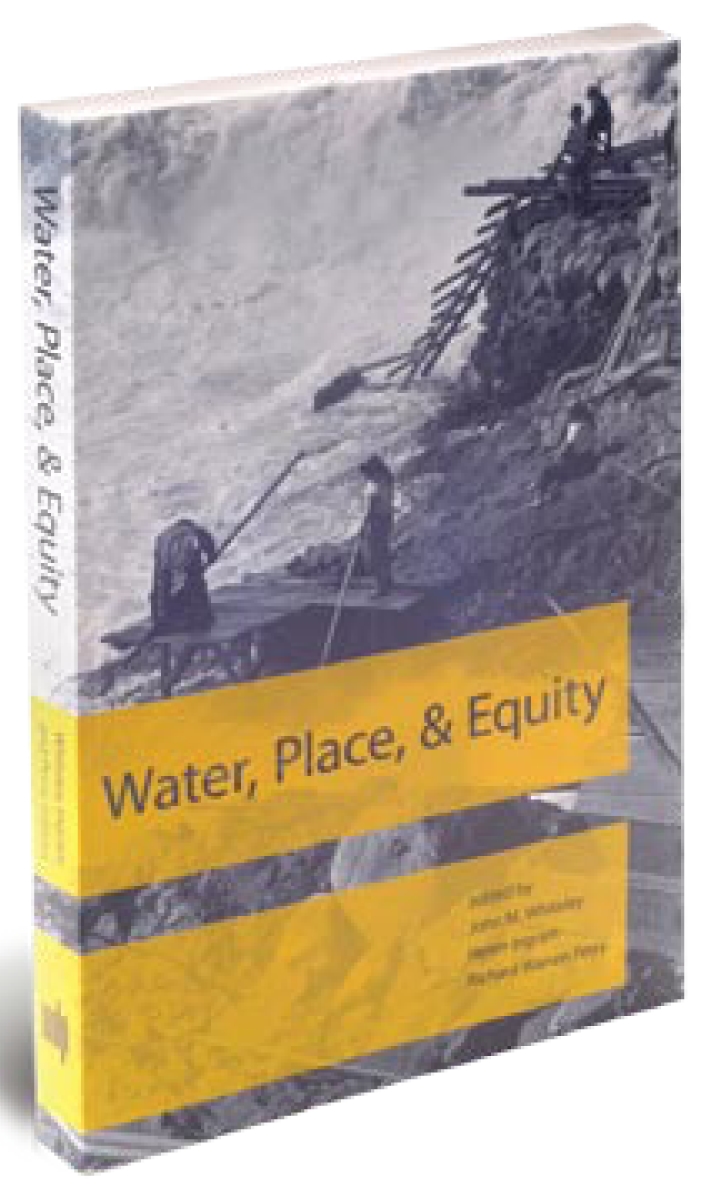# Water, Place, and Equity

**Published:** 2009-08

**Authors:** CARL BAUER

**Affiliations:** *Carl Bauer is associate professor of geography at the University of Arizona, where he teaches and does research about comparative and international water law, policy, and political economy. He has published two books about water rights and markets in Chile, the most recent entitled*Siren Song

The global water crisis has gotten wider public and political attention since the early 1990s. Growing demands for water have increased competition, conflict, and environmental impacts, so good water supplies have become relatively more scarce and valuable. Calls for policy reforms to improve how we allocate and manage water have led to disagreement on the approach those reforms should take. One core issue has been the tension between social equity and economic efficiency, with major consequences for environmental health and sustainable development.

This new book is a valuable contribution to the international water policy debate. Academics and policy analysts are the primary audiences. The editors’ basic argument is that water policies have been dominated by issues of markets and efficiency, and should be counterbalanced by a stronger emphasis on equity, fairness, and justice. The book raises “equity to its proper place as equal to efficiency among criteria to evaluate water-related actions and policies” by looking at eight case studies (seven from the Americas and one from Spain). The intellectual goal is to think through what equity involves rather than simply to praise it. The book grew out of a 2004 conference honoring political scientist Helen Ingram, whose lifelong work on water politics and water equity is well known to most people in the field.

The editors’ introduction ranges from historical roots of equity to political theory to contemporary water policy and political conflicts. The core of the argument is a critique of efficiency and the “efficiency framework,” both considered synonymous with pro-market economics. According to the editors, the “movement for water governance reform” has been dominated by proponents of markets. Anyone familiar with recent water policy debates, especially international debates, will recognize what the editors are talking about.

The editors argue that the basic flaw of efficiency is its narrow concept of value: Monetary and commodity values are the only ones that really count. This “failure to recognize value pluralism in relation to water” is a theme running through most of the book’s chapters. The authors demonstrate the importance of grappling with economic, social, political, cultural, and environmental values in water conflicts. Another theme is the idea that equity involves two things that overlap but nonetheless are not the same: fairness in process and fairness in distribution. Distributional equity is an outcome more than a process and includes who pays the costs and who enjoys the benefits.

The eight case studies are divided into two parts, one about place and one about governance, a distinction that seems unimportant to the book’s argument. All are solid, empirical, and substantive. The authors, with different analytical and disciplinary approaches, examine different specific regions and water issues. The chapters are diverse and somewhat uneven but the overall result contributes to the book’s success in breadth and depth. Topics include the “moral economy of water” in rural Colorado and external threats to local water supplies; ethical issues and the uneven distribution of costs and benefits of stormwater runoff policies in coastal California; privatization and neoliberal policies in northern Mexico and the impacts on the rural poor; the history of unequal relations between the United States and Mexico over shared rivers and boundaries; transboundary rivers in the Pacific Northwest and Canada, comparing approaches to hydropower development and fisheries management; the recent conflict over water privatization in Cochabamba, Bolivia, and the World Bank’s role in water policies; the changing significance of water uses in the historical development of mountainous Catalonia, in Spain; and the tension between technocratic authority and stakeholder participation in Brazil.

*Water, Place, and Equity* meets the authors’ twin goals of highlighting the importance of equity in water policy and exploring its complexities and different aspects. I will use the book in teaching graduate students. Its main weakness, for me, is that the case for equity often rests on a one-sided and superficial critique of efficiency, and indeed of economics in general. In their efforts to counterbalance the dominance of markets and efficiency, the editors make them too easy a target, and several chapters treat all economic analysis as synonymous with markets and commoditization. The book thus reproduces the same dichotomy of efficiency versus equity that has helped give economists so much influence in public policy. A better strategy might be to show how efficiency and equity are entangled with each other, and thereby try to reclaim economics as a field whose end is to improve the human condition, in water policy as in everything else.

## Figures and Tables

**Figure:**